# Spinal Metastasis Causing Dropped Head Syndrome in the Chiropractic Office: A Case Report

**DOI:** 10.7759/cureus.34796

**Published:** 2023-02-09

**Authors:** Eric Chun-Pu Chu, Robert J Trager, Wai Ting Lee, Damien Ming Yan Tam, Ronald Kwok

**Affiliations:** 1 New York Chiropractic and Physiotherapy Centre, New York Medical Group, Kowloon, HKG; 2 Chiropractic, Connor Whole Health, University Hospitals Cleveland Medical Center, Cleveland, USA

**Keywords:** prostate cancer, neoplasms, metastasis, neck muscles, kyphosis, chiropractic

## Abstract

Dropped head syndrome is a rare disorder involving an inability to hold the head upright. It may be caused by a variety of neuromuscular disorders and occasionally by pathological vertebral fractures.

A 79-year-old man presented to a chiropractor with a two-year history of gradual-onset chin-on-chest posture and increased thoracic kyphosis, which had failed to respond to physical therapy. The chiropractor ordered whole spine computed tomography which revealed extensive mixed lytic and sclerotic changes and multiple thoracic compression fractures suggestive of metastasis. The chiropractor promptly referred the patient to an oncologist, who performed a biopsy confirming prostate adenocarcinoma. The patient’s health deteriorated, and he expired three weeks later.

This case highlights that chiropractors should be aware that patients may present to their office with symptoms related to undiagnosed cancer, such as spinal deformity and dropped head syndrome. Chiropractors should order advanced imaging when patients have red flag signs or symptoms (e.g., older age, progressive symptoms despite care) and refer to an oncologist when clinical features or testing are suggestive of metastasis.

## Introduction

Metastatic disease commonly affects the spinal column and may cause pathological vertebral fractures, leading to pain, abnormal posture, and neurological sequelae such as weakness or impaired balance [1]. Dropped head syndrome is a rare disorder characterized by a severe kyphotic deformity of the cervicothoracic spine in both sitting and standing positions [2]. The condition is typically seen in older adults and may be caused by a variety of conditions, including myopathy, spondylosis, spinal cord injury, and occasionally by vertebral fracture, which may be related to underlying spinal metastasis [3,4]. Accordingly, providers who manage spinal complaints must be aware of dropped head syndrome, as it may be indicative of serious pathology.

A systematic review including 129 patients presenting for treatment of dropped head syndrome found that the mean age of patients was 64 years (95% CI: 61-66) [3]. The study reported that the most common causes of dropped head syndrome were isolated neck extensor myopathy (32%), Parkinson’s disease (20%), myasthenia gravis (12%), and amyotrophic lateral sclerosis (7%). Some cases of dropped head syndrome were noted to result from radiotherapy (4%) [3], as this may be a late side-effect of the treatment for head and neck cancers [5]. The review only listed a neoplastic cause of dropped head syndrome in 1% of cases [3]. More recently, a limited number of case reports and series have described vertebral metastasis and/or fracture as etiology, suggesting that these are rare yet important causes of dropped head syndrome [4,6].

Chiropractors are portal-of-entry healthcare providers who often evaluate patients with spinal disorders. As such, chiropractors may occasionally be faced with unusual case presentations which could be harbingers of serious underlying systemic disease [7]. Therefore, early identification of metastasis is imperative not only to facilitate oncologic care but also to avoid any chiropractic treatment-related fractures among patients with compromised bone integrity [8]. Despite it being important for chiropractors to identify metastasis, there is limited research on this topic [9,10].

Chiropractors are trained to recognize red flags (i.e., warning signs and symptoms) that may be indicative of serious pathology such as cancer. Examples of red flags include older age, trauma, or a focal neurologic deficit with disabling or progressive symptoms [7,11]. In such cases, chiropractors should order imaging and/or refer such patients for appropriate management without delay [7,11].

We searched the literature on January 27, 2023, via PubMed, Google Scholar, and the Index to Chiropractic Literature, using the terms “dropped head syndrome,” and "chiropractor," and recent review articles [9,10,12]. We only identified one case report describing a patient with dropped head syndrome presenting to a chiropractor, which was caused by myopathy rather than metastasis [13]. Given the limited research on this topic, we describe an elderly man with dropped head syndrome who presented to a chiropractor and was diagnosed with spinal metastasis.

## Case presentation

Patient information

A 79-year-old male with a past medical history of degenerative cervical spondylosis presented to a chiropractor with a two-year history of an inability to hold his head upright and stand up straight. He noted that his symptoms started gradually as fatigue of the neck and upper back. He also noted poor balance and required the use of a cane and assistance from a family member to ambulate. The patient noted having only mild neck and back pain, rated 1/10 on the numeric pain rating scale, and denied having any radiating pain, paresthesia, or numbness in the upper and lower limbs. He endorsed a 10-year history of nocturnal urinary urgency, however, his past serum prostate-specific antigen (PSA) test was reportedly within normal limits. Until two years prior he had been healthy, and he currently only took over-the-counter non-steroidal anti-inflammatory drugs for pain relief as needed. His World Health Organization Quality of Life score was 80%.

Six months prior to presenting to the chiropractor, the patient visited his family physician for similar but slightly less severe symptoms. The provider referred him to a physiotherapist for muscle-strengthening exercises. However, his posture deteriorated regardless. Given the progressive worsening of symptoms, the patient sought a chiropractor for another opinion.

Clinical findings

**Figure 1:**
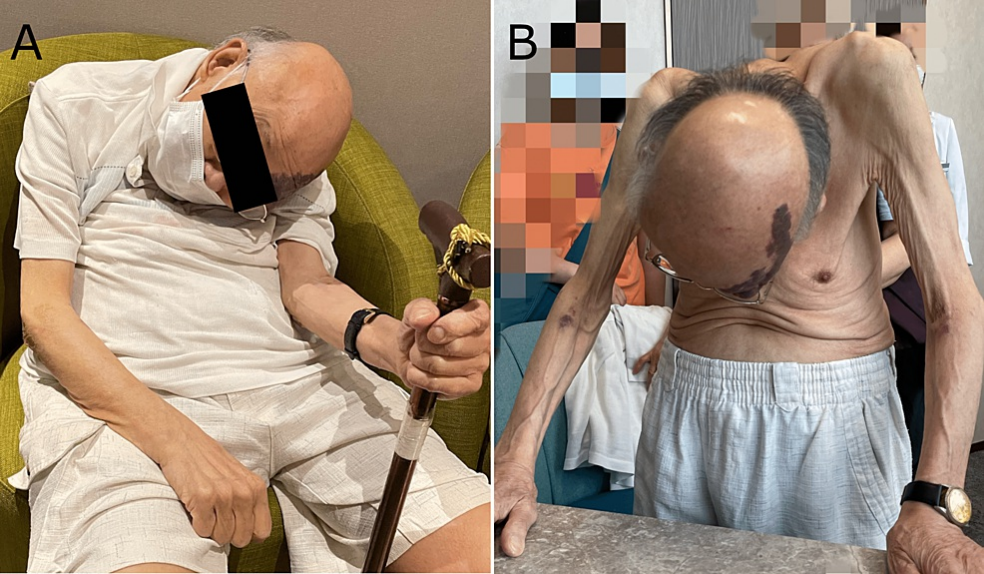
The patient demonstrated an abnormal posture. The patient had a chin-on-chest/dropped-head posture in both sitting (A) and standing (B) positions. In addition, his head was rotated to the right and laterally flexed to the left, most notably when seated.

Upon presentation, the patient was observed to have a chin-on-chest (i.e., dropped head) posture while seated and standing (Figures 1A, 1B). He used a cane to maintain balance while standing. The dropped-head posture was partially improved upon lying in a supine position. Reduced muscle tone and grating sensations were evident upon nuchal palpation. The patient’s active range of cervical extension was limited to 0°, while passive cervical extension was likewise limited (20°) and caused crepitus. Manual muscle tests of the upper limbs revealed diffuse weakness with each action graded 4 out of 5 (Medical Research Council scale). Upper and lower extremity reflex testing revealed diminished (1+) Achilles stretch reflexes bilaterally and a diminished left patellar reflex. A cranial nerve examination was normal, and no tremors, dysarthria, rigidity, hyperreflexia, or pathological reflexes (i.e., Babinski, Hoffmann) were present.

Given the patient’s worsening posture, diffuse weakness, lack of response to conservative care, and older age, the chiropractor considered thoracic spinal compression fracture as the working diagnosis, while cervical and lumbar radiculopathy were also considered. Central nervous system disorders (i.e., Parkinson’s, amyotrophic lateral sclerosis) were considered less likely given the absence of typical upper motor neuron signs. The chiropractor recommended magnetic resonance imaging (MRI) to evaluate the entire spine, however, the patient preferred to obtain computed tomography (CT) due to its reduced cost and obtained it the same week of presentation.

**Figure 2:**
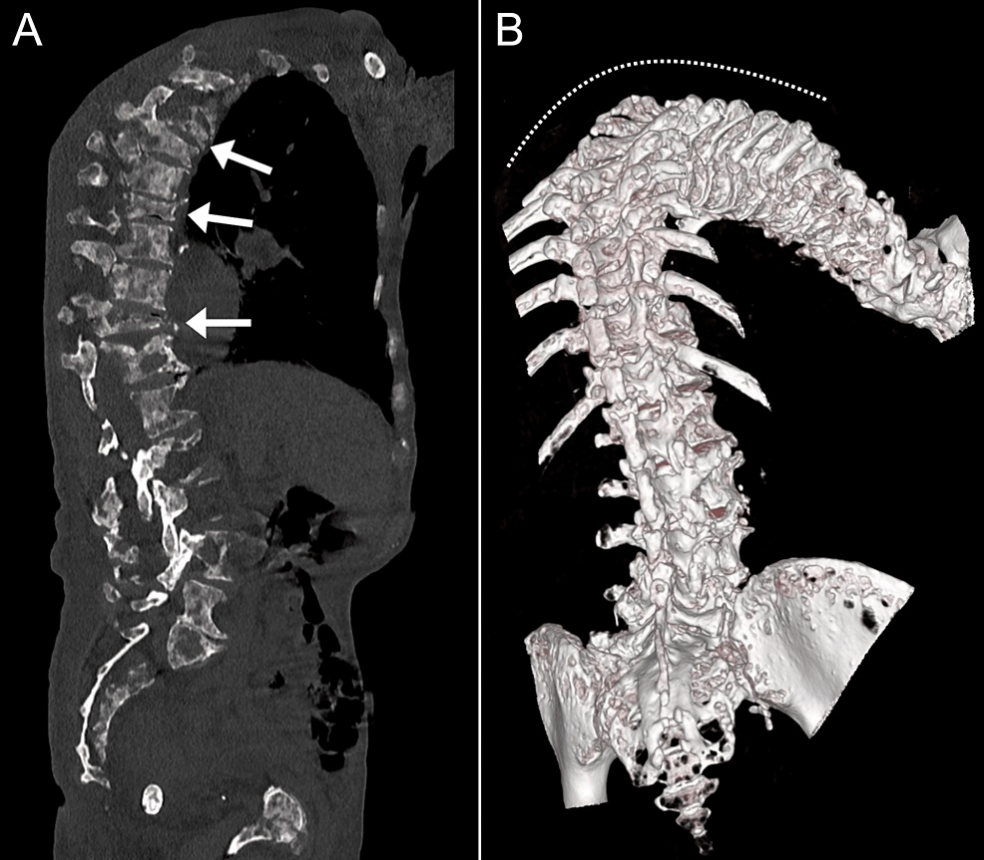
Sagittal (A) and three-dimensional reconstruction (B) of computed tomography (CT). This imaging revealed widespread mixed lytic/sclerotic changes, pathological anterior wedge compression fractures of the T5, T8, and T11 vertebral bodies (arrows), causing severe thoracic spine kyphoscoliosis (dotted line), several rib fractures (not indicated), and a grade 1 L2/L3 and L4/L5 spondylolisthesis (not indicated).

CT revealed extensive mixed lytic and sclerotic changes diffusely through the axial skeleton, including all spinal regions and several ribs, suggestive of extensive bone metastases (Figures 2A, 2B). Also evident were severe dextro-convex kyphoscoliosis of the thoracic spine, collapsed T5, T8, and T11 vertebral bodies with anterior wedging, multiple bilateral rib fractures with callus formation suggestive of old fractures (right 5th-8th, left 4th, left 7th-11th), and a grade 1 spondylolisthesis at L2/L3 and L4/L5. A mild left pleural effusion, fibrotic changes at both lung bases, and enlarged prostate were also noted. No cervical fracture was evident, while cervical spondylosis and lytic/sclerotic changes were demonstrated. Considering the extensive mixed lytic/sclerotic changes and fractures, the chiropractor considered a working diagnosis of metastasis and immediately referred the patient to an oncologist at a nearby hospital. The patient also provided written informed consent for the publication of his case and any accompanying images.

The oncologist conducted a PSA test, which was normal. However, a biopsy of the prostate confirmed a diagnosis of adenocarcinoma, leading the oncologist to make a presumptive diagnosis of prostate metastasis to the spine. One week after the chiropractic consultation, the patient was admitted to a nearby hospital under suspicion of pneumonia. MRI further supported the diagnosis of vertebral metastasis. The patient’s health rapidly deteriorated, and he passed away three weeks later.

## Discussion

This case illustrates, to our knowledge, the first published case of a patient with dropped head syndrome secondary to undiagnosed spinal metastasis presenting to a chiropractor. As CT revealed multiple collapsed thoracic vertebral bodies and other findings suggestive of metastasis, the chiropractor promptly referred the patient to an oncologist who conducted prostate biopsy and diagnosed him with metastatic adenocarcinoma of the prostate.

We suggest that the patient’s pathologic thoracic fractures and severely increased kyphosis were the primary cause of his dropped head syndrome. Previous studies have likewise reported that thoracic spine fractures causing a kyphotic deformity led to dropped head syndrome [6,14]. In one prior case series, the dropped head syndrome was reversed upon surgical correction of the thoracic kyphosis, leading the authors to suggest that the thoracic kyphosis caused stress on the cervical extensor muscles and impaired their function [6]. We suspect that this mechanism was also involved in our present case. The two primary cervical extensor muscles (semispinalis cervicis and capitis) take origin from as low as the mid-thoracic spine [5]. Such muscles, if stretched by a thoracic hyperkyphosis, could develop passive insufficiency wherein they are unable to function efficiently. Another mechanism that could have contributed to the current patient’s dropped-head posture is the paraneoplastic syndrome of cancer-associated muscle weakness [15].

This current case reinforces the diagnostic challenges of prostate cancer in men. While individuals with prostate cancer may have minimal symptoms, 19% of those with newly diagnosed prostate cancer already have bone metastases [16]. These patients are typically older males (i.e., >60 years) and may present with back pain (93% of cases), nerve root pain (66%), neurologic deficits (25%), or bladder dysfunction (3%) [16]. Providers should also be vigilant to detect any red flag signs or symptoms, such as progressively worsening neurological deficits, or symptoms that fail to improve with conservative care [17]. In addition, chiropractors should be aware that men with undiagnosed prostate cancer may rarely present in their practice, as it is a common cause of spinal metastasis [18]. For investigating suspected malignancy via imaging, CT and MRI are preferred over radiography [19].

PSA testing is often used as part of the workup for suspected prostate cancer [20]. However, as illustrated in the current case, the patient suffered from prostate cancer without having an elevated (abnormal) PSA. This may be explained in that the PSA test has a sensitivity of only 93% even when patients are symptomatic [20]. Therefore, providers should be aware that a normal PSA test does not guarantee the absence of prostate cancer and should conduct further examination if they suspect malignancy.

A previous case reported a 72-year-old male with dropped head syndrome presenting to a chiropractor caused by myopathy [13]. Similarly, both the current and previous patients were elderly men who had a poor response to medication(s) and physical therapy. However, as the previous case showed improvement after chiropractic spinal manipulation (i.e., a manual therapy applied to the spine), the authors suggested that this treatment restored the function of involved joints and muscles [13]. In contrast, spinal manipulation was contraindicated in the current case due to the presence of spinal metastasis [8]. Instead, the priority was to refer the patient for oncologic management.

The current case has certain limitations. While the findings were supported by extensive clinical evidence, imaging, and prostate biopsy results, we lacked electrodiagnostic testing and muscle biopsy which may have better characterized the patient’s dropped head syndrome. However, these tests would not have been practical to obtain given the urgent priority of oncologic management. We were unable to describe the patient’s previous primary care, physiotherapy, and PSA testing in greater detail due to a lack of supporting records. In addition, we were unable to obtain the patient’s spinal MRI results and records from the outside hospital where he was admitted and expired. Availability of this imaging could have allowed us to better characterize or rule out any potential radiculopathy or myelopathy. Additionally, we could not ascertain the patient’s specific cause of death. While the patient’s clinical presentation can be described as dropped head syndrome (i.e., chin-on-chest position with cervicothoracic kyphosis and cervical extensor weakness), his posture could be more broadly described as severe/extreme kyphosis [2,3,5]. The generalizability of this case may be limited, as chiropractors in certain regions may not have access to advanced imaging and might instead refer the patient immediately for further evaluation.

## Conclusions

We describe an elderly male presenting to a chiropractor with dropped head syndrome, ultimately diagnosed as having prostate cancer metastasis to the spine. As dropped head syndrome can be caused by serious pathology, including vertebral fracture related to underlying malignancy, providers that manage spinal complaints should be familiar with this condition and conduct necessary investigations when indicated. When testing or imaging is suggestive of malignancy, providers should promptly refer the patient to an oncologist.
